# Safety of BNT162b2 and CoronaVac during pregnancy on birth outcomes and neonatal mortality: a cohort study from Brazil

**DOI:** 10.1093/ije/dyad120

**Published:** 2023-09-10

**Authors:** Pilar Tavares Veras Florentino, Thiago Cerqueira-Silva, Luciana Freire De Carvalho, Flávia Jôse Oliveira Alves, Vinicius De Araújo Oliveira, Gislani Mateus Oliveira Aguilar, Rodrigo De Sousa Prado, Daniel Soranz, Neil Pearce, Viviane Boaventura, Guilherme Loreiro Werneck, Gerson Oliveira Penna, Mauricio Lima Barreto, Márcio Henrique De Oliveira Garcia, Manoel Barral-Netto, Enny Santos da Paixão

**Affiliations:** Centro de Integração de Dados e Conhecimentos para Saúde, Instituto Gonçalo Moniz, Fundação Oswaldo Cruz, Salvador, Brazil; Centro de Integração de Dados e Conhecimentos para Saúde, Instituto Gonçalo Moniz, Fundação Oswaldo Cruz, Salvador, Brazil; Centro de Inteligência Epidemiológica, Secretaria Municipal de Saúde do Rio de Janeiro, Rio de Janeiro, Brazil; Instituto de Estudos em Saúde Coletiva, Universidade Federal do Rio de Janeiro, Rio de Janeiro, Brazil; Centro de Integração de Dados e Conhecimentos para Saúde, Instituto Gonçalo Moniz, Fundação Oswaldo Cruz, Salvador, Brazil; Centro de Integração de Dados e Conhecimentos para Saúde, Instituto Gonçalo Moniz, Fundação Oswaldo Cruz, Salvador, Brazil; Centro de Inteligência Epidemiológica, Secretaria Municipal de Saúde do Rio de Janeiro, Rio de Janeiro, Brazil; Centro de Inteligência Epidemiológica, Secretaria Municipal de Saúde do Rio de Janeiro, Rio de Janeiro, Brazil; Câmara dos Deputados, Câmara Legislativa do Distrito Federal, Congresso Nacional, Brasília, Brazil; Faculty of Epidemiology and Population Health, London School of Hygiene and Tropical Medicine, London, UK; Laboratório de Medicina e Saúde Pública de Precisão, Instituto Gonçalo Moniz, Fundação Oswaldo Cruz, Salvador, Brazil; Faculdade de Medicina, Universidade Federal da Bahia, Salvador, Brazil; Instituto de Estudos em Saúde Coletiva, Universidade Federal do Rio de Janeiro, Rio de Janeiro, Brazil; Centro de Medicina Tropical, Universidade de Brasília, Escola do Governo de Brasília—Fiocruz, Brasília, Brazil; Centro de Integração de Dados e Conhecimentos para Saúde, Instituto Gonçalo Moniz, Fundação Oswaldo Cruz, Salvador, Brazil; Departamento de Emergências em Saúde Pública, Secretaria de Saúde, Ministério da Saúde, Brasília, Brazil; Laboratório de Medicina e Saúde Pública de Precisão, Instituto Gonçalo Moniz, Fundação Oswaldo Cruz, Salvador, Brazil; Faculdade de Medicina, Universidade Federal da Bahia, Salvador, Brazil; Laboratório de Medicina e Saúde Pública de Precisão, Instituto Gonçalo Moniz, Fundação Oswaldo Cruz, Salvador, Brazil

**Keywords:** Pregnancy, birth outcomes, COVID-19 vaccines

## Abstract

**Background:**

COVID-19 vaccines have been shown to protect pregnant individuals against mild and severe COVID-19 outcomes. However, limited safety data are available for inactivated (CoronaVac) and mRNA (BNT162b2) vaccines during pregnancy regarding their effect on birth outcomes and neonatal mortality, especially in low- and middle-income countries.

**Methods:**

We conducted a retrospective population-based cohort study in Rio de Janeiro, Brazil, with 17 513 singleton live births conceived between 15 May 2021 and 23 October 2021. The primary exposure was maternal vaccination with CoronaVac or mRNA BNT162b2 vaccines and sub-analyses were performed by the gestational trimester of the first dose and the number of doses given during pregnancy. The outcomes were pre-term birth (PTB), small for gestational age (SGA), low birthweight (LBW), low Apgar 5 and neonatal death. We used the Cox model to estimate the hazard ratio (HR) with a 95% CI and applied the inverse probability of treatment weights to generate adjusted HRs.

**Results:**

We found no significant increase in the risk of PTB (HR: 0.98; 95% CI 0.88, 1.10), SGA (HR: 1.09; 95% CI 0.96, 1.27), LBW (HR: 1.00; 95% CI 0.88, 1.14), low Apgar 5 (HR: 0.81; 95% CI 0.55, 1.22) or neonatal death (HR: 0.88; 95% CI 0.56, 1.48) in women vaccinated with CoronaVac or BNT162b2 vaccines. These findings were consistent across sub-analyses stratified by the gestational trimester of the first dose and the number of doses given during pregnancy. We found mild yet consistent protection against PTB in women who received different vaccine platforms during the third trimester of pregnancy (any vaccines, HR: 0.78; 95% CI 0.63, 0.98; BNT162b2, HR: 0.75; 95% CI 0.59, 0.99).

**Conclusions:**

This study provides evidence that COVID-19 vaccination in all trimesters of pregnancy, irrespective of the vaccine type, is safe and does not increase the risk of adverse birth outcomes or neonatal deaths.

Key Messages

- COVID-19 vaccination during pregnancy is safe against adverse birth outcomes.
- A first dose of BNT162b2 given during the third gestational trimester was mildly protective against pre-term birth.
- COVID-19 vaccines are safe against neonatal mortality (≤28 days).

## Introduction

Pregnant women have a higher risk of COVID-19-related complications, including hospitalization, need for mechanical ventilation and death, compared with the non-pregnant population of reproductive age.^1^^,^^2^ Moreover, maternal infection with SARS-CoV-2 has been associated with an increased risk of adverse fetal outcomes, including pre-term birth (PTB) and stillbirth.^3–5^ Although pregnant women were not included in pre-authorization trials of COVID-19 vaccines, vaccination is recommended for this vulnerable group in many countries. There is considerable interest in establishing the safety and effectiveness of COVID-19 vaccines in this population with real-world data.

Vaccines offer protection against mild and severe COVID-19 outcomes during pregnancy.^6^^,^^7^ Additionally, there is evidence that COVID-19 vaccination in pregnancy does not increase the risk of adverse pregnancy outcomes.^8^ However, safety studies were restricted to mRNA vaccines and conducted in high-income settings. Inactivated vaccines such as CoronaVac have been endorsed by the World Health Organization and are available for pregnant women, mainly in low- and middle-income countries.^9^ Therefore, the lack of data from low- and middle-income settings on the safety of COVID-19 vaccines in pregnancy represents a critical point in addressing vaccine hesitance.

In this study, we leveraged population-based linked data from Brazil to investigate whether there was an association between the use of COVID-19 mRNA and inactivated vaccines during pregnancy and adverse birth outcomes [pre-term births, low birthweight, small for gestational age (SGA) at birth and low Apgar score] and neonatal deaths.

## Methods

### Study setting

Rio de Janeiro municipality has 6.8 million inhabitants. In 2021, the number of pregnancies that resulted in live-born babies was 68 583. Since the beginning of the pandemic, Rio de Janeiro has had 98 deaths due to COVID-19 during pregnancy. The Rio de Janeiro vaccination programme against SARS-CoV-2 for pregnant women ≥18 years old without comorbidities began on 7 June 2021, during the Delta-dominant period.^10^ The recommended vaccines for these women were CoronaVac (Sinovac) and BNT162b2 (Pfizer-Biotech). A booster dose was recommended 4 months after second vaccination and most women were given BNT162b2.

### Study design

We conducted a retrospective population-based cohort study among live births of pregnant women between ≥18 and ≤49 years of age from Rio de Janeiro city (Brazil). The live birth registry was used to identify the study population and was extracted on 1 September 2022. We included live births from pregnancies with estimated conception dates between 15 May 2021 and 23 October 2021. The inclusion was based on the date of conception instead of the delivery dates to reduce fixed cohort bias.^11^ Based on a 40-week gestation, the delivery of pregnancies from 23 October 2021 would be 30 July 2022, allowing at least 28 days of follow-up post-birth. We excluded: (i) births occurring at <22 weeks, >44 weeks, or birthweight <500 g; (ii) women who gave birth to duplets or more; (iii) registry with missing information for education, prenatal appointments and parity; (iv) women vaccinated with vaccines other than BNT162b2 or CoronaVac; and (v) registry with vaccination date inconsistencies, vaccinated before pregnancies or those that did not follow the recommended schedule (e.g. three doses of CoronaVac).

We followed the RECORD reporting guidelines (Supplementary Table S1, available as Supplementary data at *IJE* online).^12^ The Brazilian National Commission in Research Ethics approved the research protocol (CAAE registration no. 63287822.0.0000.0040).

### Data source

Data were obtained from the Live Birth Information System (SINASC), National Immunisation System (SI-PNI) and Mortality Information System (SIM). The SINASC data set compiles the records from the Declaration of Live Birth—a legal document filed by health workers who assist the delivery.^13^ In this system, we acquired information about the mother, such as maternal age, education level, marital status and ethnicity. Also, in this data set, we obtained information about the pregnancy (prenatal appointments, parity, previous loss, length of gestation) and the newborn (birthweight, sex, Apgar score).

The SI-PNI contains data on all vaccines administered in Brazil. COVID-19 vaccines are administered by health services and recorded in point-of-care applications. From the SI-PNI, we extracted information on which COVID-19 vaccine was received in the first, second and booster doses. By linking these data with the SINASC, we determined whether vaccination occurred during, before or after pregnancy.

Death-related information was obtained from the Mortality Information System, which registers the death certificate—a mandatory legal document completed by the physician responsible for clinical care, an assistant or another practitioner from the institution who certifies the cause of death. In cases in which the death occurs without medical assistance, the death certificate is provided by a pathologist. In addition, we linked infants’ death events occurring <1 year after birth.

### Linkage process

Vaccination records were linked with the live birth cohort from the SINASC to identify those vaccinated during pregnancy. Maternal name, date of birth and zip code were used in the matching process. The similarity between names and neighbourhoods recorded in the SINASC and SI-PNI was compared using the Jaro-Winkler string comparator.^14^ The Jaro-Winkler string comparator counts the number of common characters between two strings and the number of transpositions of these common characters, producing similarity values varying between 1 (perfectly similar) and 0 (non-similar).^13^ We categorized the string comparator score as (0,0.85), (0.85,0.95) and (0.95,1). Then, we used a deterministic approach based on patient identifiers in three steps: (i) string comparator of >0.95 for maternal name and match on date of birth AND zip code; (ii) string comparator of >0.95 for maternal name and match on date of birth OR zip code followed by a clerical review; (iii) string comparator of <0.95 and >0.85 for maternal name and match on date of birth AND/OR zip code followed by a clerical review. This data linkage approach is designed to minimize the number of false matches.^15^ We deterministically linked the SINASC and SIM using the live-born declaration number.

For the validation process, we compared the number of matches in our linked data set (SIM–SINASC) with the number from the official municipality statistics. We obtained a sensitivity of ∼92% for the linkage between the SINASC and the SIM.

### Exposures and covariates

Our exposure of interest is vaccination during pregnancy. Vaccinated women were defined as those who received their first BNT162b2 or CoronaVac dose ≥14 days post-conception, ≤3 days before the delivery. Additional doses during pregnancy were also considered ≤3 days before birth. These cut-offs were used to reduce potential misclassification of pre-pregnancy or post-partum vaccination during pregnancy.

We adjusted the model using the inverse probability weights based on the propensity-for-vaccination model built on a priori selected covariates.^16^ The covariates included the mother's age, number of prenatal appointments (1–3, 4–6 or ≥7), education (0–3, 4–7, 8–11 or ≥12 years), parity (multiparous or nulliparous), previous stillbirth, marital status (married, single, common-law marriage or other) and race (White, Black, mixed race or others—Asian and Indigenous).

### Outcomes

We defined PTB as a live birth before 37 weeks. SGA at birth was established using an intergrowth scale in singleton live-born infants below the 10th centile of the sex-specific birthweight-for-gestational-age distribution.^17^ Low birthweight (LBW) was defined as infants weighing <2.5 kg. We also accessed the Apgar 5 (<7) score—a standardized test performed 5 min after birth to check how well the baby tolerated the birth process.

The risk window for PTB was from 22 to 37 weeks of gestation (pregnancy day 258). In the case of LBW or SGA, it was 22 weeks to the end of pregnancy and, for neonatal mortality, it was within 28 days of the birth date.

### Statistical analyses

Vaccination was considered a time-varying exposure, which means that women contributed to the cohort as unexposed (before vaccination) and exposed (after vaccination). Robust sandwich variance estimation was used to account for statistical dependence across repeated observations because of changes in vaccination status. Gestational age in days was used as the timescale; ongoing pregnancies on 15 May 2021 started contributing the time from that point and followed up until either the event occurrence or censoring at the end of the outcome-specific period.

We used the Cox model to estimate the hazard ratio (HR) with a 95% CI and applied the inverse probability of treatment weights to generate the adjusted HR (aHR). The weights to be vaccinated were estimated using generalized boosted regression trees with the selected covariates representing the predicted probability of being vaccinated during pregnancy. Then the inverse probability of treatment weights was used to balance the groups across the baseline covariates regarding the likelihood of receiving at least one dose during the pregnancy. The weights were truncated at 1.0 and 99.0%ile. We assessed the proportional hazards assumption using a test based on Schoenfeld residuals for the main analysis and not by trimester of the first dose stratified by vaccine type (CoronaVac or BNT162b2) (Supplementary Table S2, available as Supplementary data at *IJE* online).^18^

In the primary analysis, we treated vaccination status as receiving one or more doses of CoronaVac or BNT162b2 during pregnancy. The secondary analysis consisted of stratifying by vaccine type, number of doses (up to three) and by trimester of their first dose during pregnancy. The timings for each trimester were: first (14–97 days), second (98–195 days) and third (196 up to delivery). Moreover, the analyses by trimester were also stratified by first-dose vaccine type (CoronaVac or BNT162b2).

We conducted a sensitivity analysis to evaluate the effect of subsequent doses during pregnancy in already primed women. We included unvaccinated women and those who received at least one dose before the pregnancy and at least one dose during the pregnancy. We used a non-parametric bootstrapping procedure with 500 iterations to calculate the bias-corrected 95% CIs for all estimates. All data processing and analyses were done by using R (version 4.1.1) using the tidyverse, survival, WeightIt and bcaboot packages.^19^

## Results

**Figure 1.**
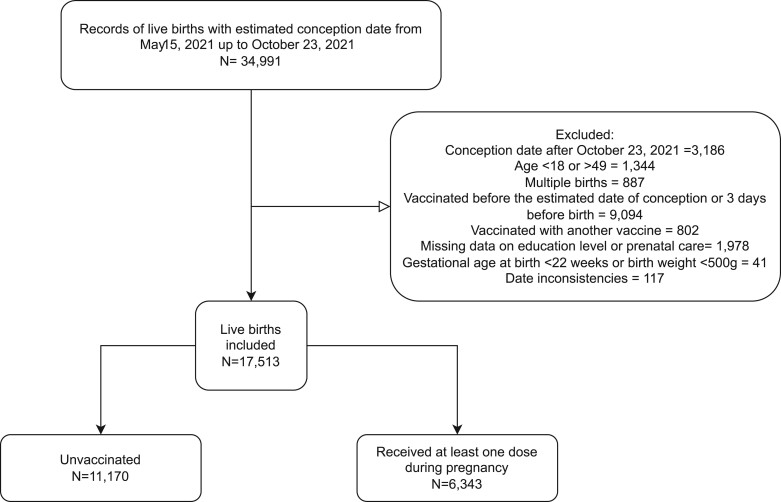
Selection of study participants

**Figure 2.**
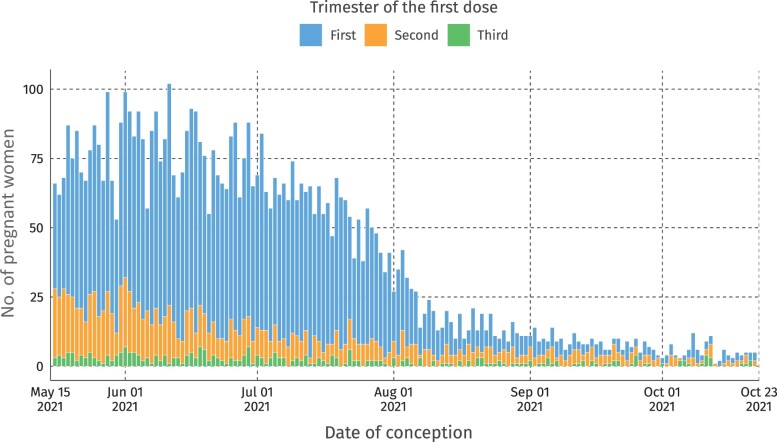
Distribution of pregnant women vaccinated by date of conception and stratified by trimester

After exclusion, we analysed 17 513 live births during the study period, of whom 11 170 (63.8%) were not vaccinated and 6343 (36.2%) had received at least one dose of BNT162b2 or CoronaVac during pregnancy (Figure 1). Most of those women who were vaccinated during pregnancy finished their primary schema (two doses), namely 3874 (62%), and 1176 (18.4%) received a booster dose during pregnancy. Most women were vaccinated in the first trimester of pregnancy (Figure 2).

The median age was 26 years (interquartile 22–33) for vaccinated and 29 years (interquartile 24–34) for unvaccinated woman. The distribution according to prenatal appointments, parity and race was similar between unvaccinated and vaccinated pregnant women (Table 1). However, married women represented only 18% of those vaccinated vs 41% among those unvaccinated. Furthermore, women with >12 years of education were less frequent (17%) among the vaccinated compared with those unvaccinated (34%).

**Table 1.** Pregnant women participants characteristics

Characteristic	Unvaccinated (*n*=11 170)	Vaccinated (*n*=6343)	Unweighted SMD	Weighted SMD
Age group (years)				
18–24	3005 (27%)	2637 (42%)	0.15	0.02
25–29	2931 (26%)	1742 (27%)	0.01	<0.01
30–34	2693 (24%)	1180 (19%)	0.06	0.01
35–49	2541 (23%)	784 (12%)	0.1	0.01
Age (years), median (IQR)	29 (24–34)	26 (22–31)	0.38	0.05
Number of prenatal appointments				
None	128 (1.1%)	38 (0.6%)	0.01	<0.01
1–3	452 (4.0%)	241 (3.8%)	<0.01	<0.01
4–6	1660 (15%)	962 (15%)	<0.01	<0.01
≥7	8930 (80%)	5102 (80%)	<0.01	0.01
Years of schooling				
0–3	96 (0.9%)	72 (1.1%)	<0.01	<0.01
4–7	1114 (10%)	914 (14%)	0.04	0.01
8–11	6158 (55%)	4310 (68%)	0.13	0.02
≥12	3802 (34%)	1047 (17%)	0.18	0.03
Nulliparous	4173 (37%)	2395 (38%)	<0.01	<0.01
Previous stillbirth	2428 (22%)	1329 (21%)	0.01	<0.01
Marital status				
Married	4532 (41%)	1139 (18%)	0.23	0.04
Single	5767 (52%)	4854 (77%)	0.25	0.04
Common-law marriage	542 (4.9%)	231 (3.6%)	0.01	<0.01
Others	329 (2.9%)	119 (1.9%)	0.01	<0.01
Race				
White	4141 (37%)	1721 (27%)	0.1	0.01
Mixed race	5071 (45%)	3307 (52%)	0.07	0.01
Black	1673 (15%)	1178 (19%)	0.04	<0.01
Other	285 (2.6%)	137 (2.2%)	<0.01	<0.01
Vaccination status at birth				
One dose				
BNT162b2	–	1138 (18%)		
CoronaVac	–	155 (2.4%)		
Two doses				
2 × BNT162b2	–	3081 (49%)		
2 × CoronaVac	–	793 (13%)		
Three doses				
3 × BNT162b2	–	534 (8.4%)		
2 × CoronaVac + 1×BNT162b2	–	642 (10%)		
Unvaccinated	11 170 (100%)	–		
Outcomes				
Small for gestational age	725 (6.5%)	474 (7.5%)		
Low weight (<2.5 kg)	1019 (9.1%)	528 (8.3%)		
Pre-term birth (<37 weeks)	1320 (12%)	677 (11%)		
Apgar <7^a^	110 (1.0%)	50 (0.8%)		
Neonatal death (≤28 days)	76 (0.7%)	36 (0.6%)		

a Missing data (133 unvaccinated and 86 vaccinated).

IQR, interquartile range; SMD, standardized mean difference.

A total of 677 (10.7%) live births from vaccinated women were pre-term, 474 (7.5%) were SGA, 528 (8.3%) had LBW and 50 (0.8%) presented with low Apgar 5 compared with 1320 (12%) PTB, 725 (6.5%) SGA, 1019 (9.1%) LBW and 110 (1.0%) low Apgar 5 among unvaccinated women, respectively (Table 1).

**Figure 3.**
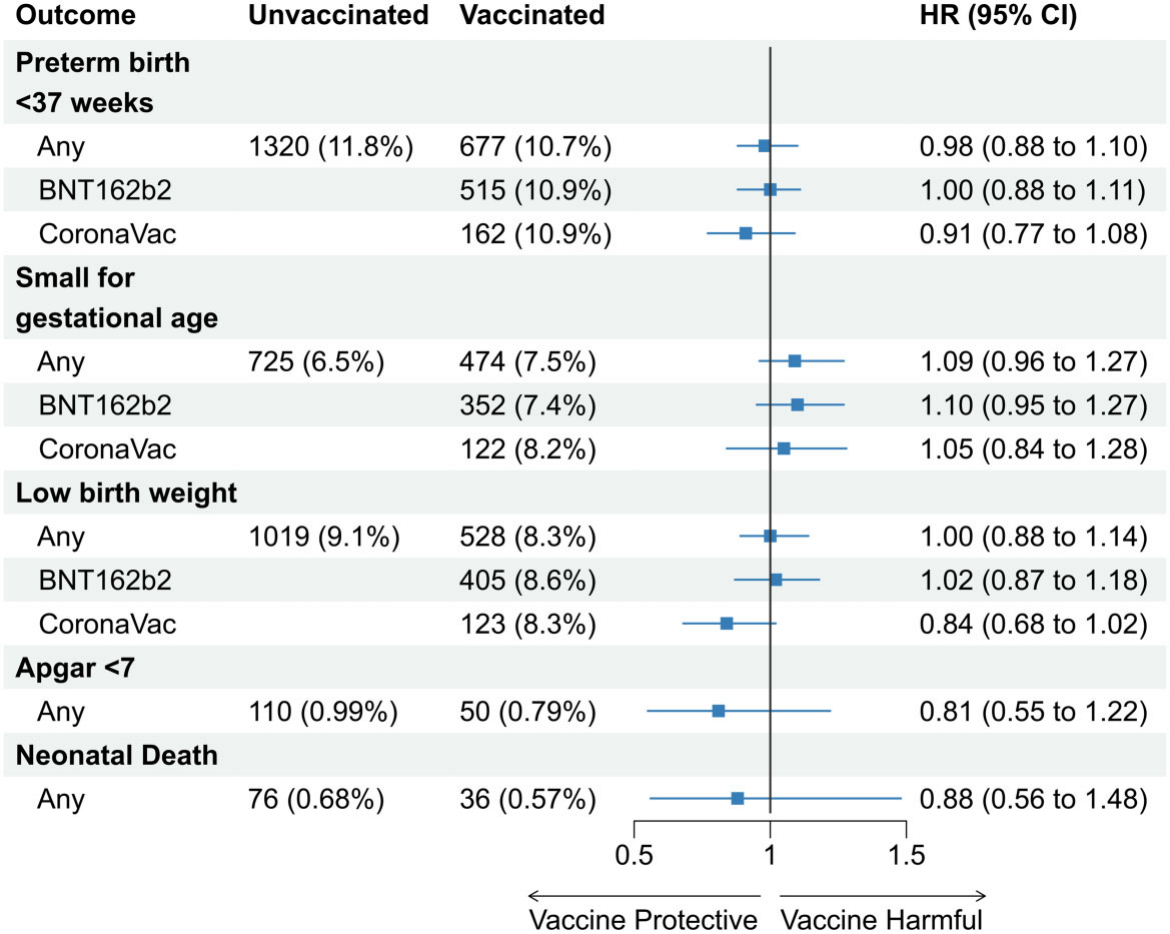
Hazard ratios for neonatal adverse outcomes among women vaccinated with at least one dose of BNT162b2 or CoronaVac

There was no association between COVID-19 vaccination during pregnancy and PTB (HR: 0.98; 95% CI 0.88, 1.10), SGA (HR: 1.09; 95% CI 0.96, 1.27), LBW (HR: 1.00; 95% CI 0.88, 1.14) or low Apgar 5 (HR: 0.81; 95% CI 0.55, 1.22) (Figure 3). In subgroup analyses, for women vaccinated with at least one dose in the first and second trimesters, the risk of PTB was 0.96 (95% CI 0.76, 1.21) and 1.05 (95% CI 0.93, 1.19), respectively. However, women vaccinated with at least one dose of any vaccine 0.78 (95% CI 0.63, 0.98) or BNT162b2 0.75 (95% CI 0.59, 0.99) in the third trimester had slightly decreased risk of PTB (Supplementary Table S3, available as Supplementary data at *IJE* online). Furthermore, no association was observed between vaccination during pregnancy and the remaining birth outcome (SGA and LBW) in subgroup analyses by trimester, the number of doses or the vaccine scheme (Supplementary Table S3, available as Supplementary data at *IJE* online).

There were 36 (0.6%) neonatal deaths among live births from vaccinated women and 76 (0.7%) from unvaccinated women. Vaccination with any vaccine was not associated with the risk of neonatal death (HR: 0.88; 95% CI 0.56, 1.48).

Finally, we also investigated the women who received at least one dose of the COVID-19 vaccine before and during the pregnancy. Results from this sensitivity analysis were similar to those from the main analysis (Supplementary Table S4, available as Supplementary data at *IJE* online).

## Discussion

Our study found that vaccination with BNT162b2 or CoronaVac during pregnancy did not increase the risk of PTB, SGA, LBW, low Apgar score in newborns or neonatal death (<28 days). The results were similar across the subgroups, by trimester of the first dose and by the number of doses given during gestation, except for PTB. Specifically, the data indicated that receiving the BNT162b2 vaccine during the third trimester was associated with a reduced risk of PTB, whereas the CoronaVac vaccine did not show a significant association with this outcome.

Studies have estimated the risk of mRNA vaccines during pregnancy for adverse pregnancy outcomes.^20–22^ However, there are still no data on CoronaVac safety during pregnancy. Nevertheless, our findings with the BNT162b2 and CoronaVac vaccines were similar and comparable with recent studies from different countries using mRNA vaccines. The HR for PTB and SGA among Canadian pregnant women was 1.02 (95% CI 0.96, 1.08) and 0.98 (95% CI 0.93, 1.03), respectively (Fell *et al.* 2022).^23^ Among Israeli pregnant women, no impact of BNT162b2 was observed for PTB [risk ratio *RR) = 0.95; 95% CI 0.83, 1.10] and SGA (RR = 0.97; 95% CI 0.87, 1.08).^22^ Furthermore, Scandinavian pregnant women receiving mRNA vaccines compared with unvaccinated women did not present a higher risk of PTB (0.98; 95% CI 0.91, 1.05), SGA (0.97; 95% CI 0.90, 1.04) or low Apgar newborns (0.97; 95% CI 0.87, 1.08). Moreover, we detected mild protection from PTB (HR: 0.79; 95% CI 0.64, 0.98) among women vaccinated in the third trimester with BNT162b2, consistently with a study from the USA (aHR: 0.82; 05% CI 0.72, 0.94), with any mRNA vaccine.^24^

Data on COVID-19 vaccines and neonatal death (≤28 days) are scarce. However, a study from Israel analysed infant death (≤180 days) and found no risk related to vaccination (RR: 0.84; 95% CI 0.43, 1.72).^22^ We analysed neonatal death (≤28 days) and found no increase in risk after vaccination (HR: 0.88; 95% CI 0.55, 1.40).

Our study has several strengths; first, the three databases used are universal and mandatory, preventing selection bias. Second, we used propensity scores estimated through the generalized boosted model, which is more accurate than conventional methods.^25^ Third, the selection based on conception date instead of birth date removes fixed cohort bias.^11^ Finally, all data came from one city, which implies a more homogenous COVID-19 health policy for the study population.

However, there are limitations to be accounted for. First, our data set did not include the history of COVID-19 during pregnancy. Furthermore, misclassification of the conception date may have occurred. Second, the linkage strategy used in this study to assess the exposure (vaccination) is designed to minimize the number of false matches; however, missed matches might have occurred.^15^ Therefore, women who were vaccinated during pregnancy may have been misclassified as not vaccinated (due to missed matches) and the estimated HR diluted (assuming a non-differential misclassification). Due to the small sample size, we could not estimate the HR of neonate death and low Apgar 5 segmenting by trimester, dose or vaccine scheme. Although CoronaVac has been included in the analysis and the overall data are consistent with those for BNT162b2, the sample size may not be large enough to draw robust conclusions when the data are stratified by trimester/dose. As we are using live births data, our study did not account for stillbirth, which is an important outcome to estimate vaccine safety. Also, our database presents only registers of live-born babies, so we cannot study fetal loss. Finally, missing education and prenatal data information may have influenced the estimates.

In conclusion, our study shows no evidence of an increased risk of birth or neonatal adverse outcomes in pregnant women vaccinated with BNT162b2 or CoronaVac with up to three doses. The present study, along with previous data on the effectiveness of COVID-19 vaccines during pregnancy, reinforces that vaccination is safe and protects women and newborns from adverse infection outcomes.

## Ethics approval

The Brazilian National Commission in Research Ethics approved the research protocol (CAAE registration no. 63287822.0.0000.0040).

## Supplementary Material

dyad120_Supplementary_Data

## Data Availability

The data underlying this article cannot be publicly shared. In this study, we used anonymized secondary data following the Brazilian Personal Data Protection General Law, but they are vulnerable to re-identification by third parties as they contain dates of relevant health events regarding the same person. Each member of the research team signed a term of confidentiality before accessing the data. Data were manipulated in a secure computing environment, ensuring protection against data leakage.
