# Supplementary material for: Safety of BNT162b2 and CoronaVac during pregnancy on birth outcomes and neonatal mortality: a cohort study from Brazil
Source: Int J Epidemiol. 2023 Sep 10;52(6):1708–15. doi: 10.1093/ije/dyad120 (PMC10749744; doi:10.1093/ije/dyad120)
Supplement: dyad120_Supplementary_Data [file dyad120_supplementary_data.docx]

**Supplement 1**

**Safety of BNT162b2 and CoronaVac in pregnant women: a cohort study from Brazil**

Pilar T V Florentino PhD*^1^, Thiago Cerqueira-Silva*^1,2^ PhD, Luciana Freire de Carvalho MSc*^3^, Flávia J O Alves PhD^1^, Vinicius de Araújo Oliveira MD^1^, Gislani Mateus Oliveira Aguilar MSc^3^, Rodrigo de Sousa Prado MD^3^, Daniel Soranz MD^4^, Neil Pearce PhD^5^, Viviane S. Boaventura^1,2^ PhD, Guilherme L Werneck DSc^6,7^, Gerson O Penna PhD^8^, Mauricio L Barreto MD^1^, Márcio Henrique de Oliveira Garcia MSc ^9^**†**, Manoel Barral-Netto MD^1^**†**, Enny S Paixão PhD^5^**†**

# Table S1 - STROBE/RECORD checklist

**Table S2: Proportional hazards tests based on Schoenfeld residuals.**

**Table S3. Neonatal adverse outcomes hazard ratio among vaccinated women stratified by trimester or number of doses given during pregnancy.**

**Table S4. Neonatal adverse outcomes hazard ratio in women vaccinated before pregnancy with any vaccine type and number of doses.**

# Table S1 - STROBE/RECORD checklist

|  | **Item No.** | **STROBE items** | **RECORD items** | **Location in manuscript where items are reported** |
| --- | --- | --- | --- | --- |
| **Title and abstract** | | |  |  |
|  | 1 | (a) Indicate the study’s design with a commonly used term in the title or the abstract (b) Provide in the abstract an informative and balanced summary of what was done and what was found | RECORD 1.1: The type of data used should be specified in the title or abstract. When possible, the name of the databases used should be included.  RECORD 1.2: If applicable, the geographic region and timeframe within which the study took place should be reported in the title or abstract.  RECORD 1.3: If linkage between databases was conducted for the study, this should be clearly stated in the title or abstract. | 1.1. The type of data is described in the Abstract, and details in the Methods in the section “Study setting, Study design, and data sources”  1.2. Time and municipality described in the Abstract and methods (“Study setting, Study design)  1.3. Linkage of databases pointed in the methods (“Linkage process”) |
| **Introduction** | | |  |  |
| Background rationale | 2 | Explain the scientific  background and rationale for the investigation being reported |  | Introduction Section, paragraphs 1 and 2 |
| Objectives | 3 | State specific objectives, including any prespecified hypotheses |  | Introduction Section, paragraph 3 |
| **Methods** | | |  |  |
| Study Design | 4 | Present key elements of study design early in the paper |  | Methods paragraph 1 and 2 (Study setting, Study design) |
| Setting | 5 | Describe the setting, locations, and relevant dates, including periods of recruitment, exposure, follow-up, and data collection |  | Methods paragraphs 1-6 |
| Participants | 6 | 1. Cohort study - Give the eligibility criteria, and the sources and methods of selection of participants. Describe methods of follow-up   Case-control study - Give the eligibility criteria, and the sources and methods of case ascertainment and control selection. Give the rationale for the choice of cases and controls Cross-sectional study - Give the eligibility criteria, and the sources and methods of selection of participants     1. Cohort study - For matched studies, give matching criteria and number of exposed and unexposed   Case-control study - For matched studies, give matching criteria and the number of controls per case | RECORD 6.1: The methods of study population selection (such as codes or algorithms used to identify subjects) should be listed in detail. If this is not possible, an explanation should be provided.    RECORD 6.2: Any validation studies of the codes or algorithms used to select the population should be referenced. If validation was conducted for this study and not published elsewhere, detailed methods and results should be provided.    RECORD 6.3: If the study involved linkage of databases, consider use of a flow diagram or other graphical display to demonstrate the data linkage process, including the number of individuals with linked data at each stage. | 6.1 – Informations were included in:  Methods paragraph 1-7  6.2.NA  6.3. Informations were included in:  Methods paragraph 7 and Figure1 |
| Variables | 7 | Clearly define all outcomes, exposures, predictors, potential confounders, and effect modifiers. Give diagnostic criteria, if applicable. | RECORD 7.1: A complete list of codes and algorithms used to classify exposures, outcomes, confounders, and effect modifiers should be provided. If these cannot be reported, an explanation should be provided. | Methods paragraphs 8-11 |
| Data sources/ measurement | 8 | For each variable of interest, give sources of data and details of methods of assessment (measurement).  Describe comparability of assessment methods if there is  more than one group |  | Methods paragraphs 4-6 (Data Source), Paragraphs 8-11 |
| Bias | 9 | Describe any efforts to address potential sources of bias |  | Methods paragraph 8 |
| Study size | 10 | Explain how the study size was arrived at |  | Methods paragraph 2,3,7 and Figure 1 |
| Quantitative variables | 11 | Explain how quantitative variables were handled in the analyses. If applicable, describe which groupings were chosen,  and why |  | Methods paragraph 10,11 |
| Statistical methods | 12 | (a) Describe all statistical methods, including those used to control for confounding (b) Describe any methods used to examine subgroups and interactions   1. Explain how missing data were addressed 2. Cohort study - If applicable, explain how loss to follow-up was addressed   Case-control study - If applicable, explain how matching of cases and controls was addressed  Cross-sectional study - If applicable, describe analytical methods taking account of sampling strategy   1. Describe any sensitivity analyses |  | (a) Methods paragraph 12-15  (b) Methods paragraph 15  (c) Methods paragraph 12-15  (d) Methods paragraph 12,14  (e) Methods paragraph 15 |
| Data access and cleaning methods |  |  | RECORD 12.1: Authors should describe the extent to which the investigators had access to the database population used to create the study population. | Methods paragraph 4-6 |
|  |  |  | RECORD 12.2: Authors should provide information on the data cleaning methods used in the study. | Methods paragraph 2-3 and Figure1 |
| Linkage |  |  | RECORD 12.3: State whether the study included person-level,  institutional-level, or other data linkage across two or more databases. The methods of linkage and methods of linkage quality evaluation should be provided. | Methods paragraph 7 and Figure1 |
| Participants | 13 | 1. Report the numbers of individuals at each stage of the study (e.g., numbers potentially eligible, examined for eligibility, confirmed eligible, included in the study, completing follow-up, and analysed) 2. Give reasons for nonparticipation at each stage. (c) Consider use of a flow diagram | RECORD 13.1: Describe in detail the selection of the persons included in the study (i.e., study population selection) including filtering based on data quality, data availability and linkage. The selection of included persons can be described in the text and/or by means of the study flow diagram. | Methods paragraph 1-3 and Figure 1 |
| Descriptive data | 14 | 1. Give characteristics of study participants (e.g., demographic, clinical, social) and information on exposures and potential confounders 2. Indicate the number of participants with missing data for each variable of interest (c) Cohort study - summarise follow-up time (e.g., average and total amount) |  | In the Results paragraph 1 and 2, Figures 1 and 2 |
| Outcome data | 15 | Cohort study - Report numbers of outcome events or summary measures over time  Case-control study - Report numbers in each exposure category, or summary measures of exposure  Cross-sectional study - Report numbers of outcome events or summary measures |  | In the Results paragraph 1 and 2, Figures 1 and 2 |
| Main results | 16 | (a) Give unadjusted estimates and, if applicable, confounderadjusted estimates and their precision (e.g., 95% confidence interval). Make clear which confounders were adjusted for and why they were included (b) Report category boundaries when continuous variables were categorized  (c) If relevant, consider translating estimates of relative risk into absolute risk for a meaningful time period |  | In the Results paragraphs 2-4, Figure 3 |
| Other analyses | 17 | Report other analyses done—  e.g., analyses of subgroups and interactions, and sensitivity analyses |  | Subgroups analyses are presented in the results section paragraph 4 |
| Key results | 18 | Summarise key results with reference to study objectives |  | Summarised both in abstracts and results |
| Limitations | 19 | Discuss limitations of the study, taking into account sources of potential bias or imprecision. Discuss both direction and magnitude of any potential bias | RECORD 19.1: Discuss the  implications of using data that were not created or collected to answer the specific research question(s). Include discussion of misclassification bias, unmeasured confounding, missing data, and changing eligibility over time, as they pertain to the study being reported. | In discussion section paragraph 4 |
| Interpretation | 20 | Give a cautious overall interpretation of results considering objectives, |  | Discussion paragraph 1-5 |
|  |  | limitations, multiplicity of analyses, results from similar studies, and other relevant evidence |  | Discussion paragraph 4 |
| Generalisability | 21 | Discuss the generalisability (external validity) of the study results |  | Discussion paragraph 5 |
| Funding | 22 | Give the source of funding and the role of the funders for the present study and, if applicable, for the original study on which the present article is based |  | In the section ‘Role of Funding” |
| Accessibility of protocol, raw data, and programming code |  |  | RECORD 22.1: Authors should provide information on how to access any supplemental information such as the study protocol, raw data, or programming code. | These information are available at: <https://vigivac.fiocruz.br> – except for raw data, that is protected under Brazilian personal data protection law (LGPD), as explained in Data availability section |

**Table S2: Proportional hazards tests based on Schoenfeld residuals**

|  |  |  |
| --- | --- | --- |
| **Outcome** | **p-value** | **Group** |
| Preterm Birth | 0.17 | Any vaccine |
| Preterm Birth | 0.12 | CoronaVac |
| Preterm Birth | 0.41 | BNT162b2 |
| SGA | 0.71 | Any vaccine |
| SGA | <0.01 | CoronaVac |
| SGA | 0.36 | BNT162b2 |
| LBW | 0.24 | Any vaccine |
| LBW | 0.03 | CoronaVac |
| LBW | 0.74 | BNT162b2 |
| Apgar<7 | 0.90 | Any vaccine |
| Neonatal death | 0.11 | Any vaccine |

**Table S3. Neonatal adverse outcomes hazard ratio among vaccinated women stratified by trimester or number of doses given during pregnancy.**

|  | HR (95% CI) | | |
| --- | --- | --- | --- |
| **Outcome** | **Preterm** | **SGA** | **LBW** |
| **Any Vaccine** |  |  |  |
| *Number of doses* | |  |  |
| 1 | 1.01 (0.84; 1.25) | 1.09 (0.85; 1.40) | 0.97 (0.79; 1.24) |
| 2 | 0.96 (0.86;1.09) | 1.06 (0.90; 1.25) | 1.02 (0.87; 1.18) |
| 3 | 1.04 (0.84;1.32) | 1.16 (0.93; 1.45) | 0.94 (0.72; 1.28) |
|  |  |  |  |
| *Trimester* |  |  |  |
| First | 0.96 (0.79;1.22) | 0.93 (0.69; 1.26) | 0.97 (0.73; 1.29) |
| Second | 1.05 (0.93;1.18) | 1.11 (0.94; 1.32) | 0.97 (0.85; 1.13) |
| Third | 0.78 (0.63;0.98) | 1.14 (0.94; 1.39) | 1.09 (0.93; 1.40) |
|  |  |  |  |
| **CoronaVac** |  |  |  |
| *Number of doses* | |  |  |
| 1 | 0.68 (0.40;1.30) | 0.81 (0.44; 1.56) | 0.41 (0.19; 0.91) |
| 2 | 0.88 (0.71;1.11) | 1.06 (0.83; 1.47) | 0.90 (0.70; 1.19) |
| 3 | 1.07 (0.82;1.51) | 1.11 (0.85; 1.54) | 0.88 (0.61; 1.22) |
|  |  |  |  |
| *Trimester* |  |  |  |
| First | 0.79 (0.61; 1.06) | 1.03 (0.72; 1.46) | 0.82 (0.62; 1.15) |
| Second | 1.11 (0.84; 1.54) | 0.99 (0.69; 1.49) | 0.86 (0.60; 1.26) |
| Third | 0.85 (0.58; 1.24) | 1.11 (0.85; 1.53) | 0.83 (0.60; 1.32) |
|  |  |  |  |
| **BNT162b2** |  |  |  |
| *Number of doses* | |  |  |
| 1 | 1.04 (0.87; 1.28) | 1.12 (0.86; 1.50) | 1.01 (0.83; 1.32) |
| 2 | 0.98 (0.86; 1.14) | 1.06 (0.89; 1.31) | 1.02 (0.88; 1.23) |
| 3 | 0.98 (0.66; 1.45) | 1.22 (0.88; 1.79) | 0.98 (0.64; 1.49) |
|  |  |  |  |
| *Trimester* |  |  |  |
| First | 1.10 (0.80; 1.59) | 0.85 (0.58; 1.39) | 1.06 (0.79; 1.63) |
| Second | 1.04 (0.93; 1.19) | 1.13 (0.93; 1.35) | 1.03 (0.88; 1.21) |
| Third | 0.75 (0.59; 0.99) | 1.16 (0.91; 1.45) | 0.91 (0.72; 1.29) |

**Table S4. Demographic characteristics of unvaccinated and women who received, at least one, dose of vaccine before and during pregnancy.**

| **Characteristic** | **Unvaccinated** | **Vaccinated** | **Unweighted** | **Weighted** |
| --- | --- | --- | --- | --- |
|  | **N = 11,170** | **N = 3,237** | **SMD** | **SMD** |
| **Age group- years** |  |  |  |  |
| **18-24** | **3,005 (27%)** | **834 (26%)** | **0.01** | **0.05** |
| **25-29** | **2,931 (26%)** | **839 (26%)** | **<0.01** | **0.02** |
| **30-34** | **2,693 (24%)** | **841 (26%)** | **0.02** | **0.03** |
| **35-49** | **2,541 (23%)** | **723 (22%)** | **<0.01** | **0.04** |
| **Age (years), median - interquartile range** | **29 (24, 34)** | **29 (24, 34)** | **0.04** | **0.15** |
| **Number of prenatal appointments** |  |  |  |  |
| **None** | **128 (1.1%)** | **16 (0.5%)** | **<0.01** | **<0.01** |
| **3-Jan** | **452 (4.0%)** | **78 (2.4%)** | **0.01** | **<0.01** |
| **6-Apr** | **1,660 (15%)** | **331 (10%)** | **0.04** | **0.02** |
| **≥ 7** | **8,930 (80%)** | **2,812 (87%)** | **0.07** | **0.03** |
| **Years of schooling** |  |  |  |  |
| **0 -3** | **96 (0.9%)** | **35 (1.1%)** | **<0.01** | **<0.01** |
| **7-Apr** | **1,114 (10.0%)** | **302 (9.3%)** | **<0.01** | **0.01** |
| **11-Aug** | **6,158 (55%)** | **1,976 (61%)** | **0.06** | **0.04** |
| **≥ 12** | **3,802 (34%)** | **924 (29%)** | **0.05** | **0.06** |
| **Nulliparous** | **4,173 (37%)** | **1,185 (37%)** | **<0.01** | **<0.01** |
| **Previous stillbirth** | **2,428 (22%)** | **777 (24%)** | **0.02** | **<0.01** |
| **Marital Status** |  |  |  |  |
| **Married** | **4,532 (41%)** | **835 (26%)** | **0.15** | **<0.01** |
| **Single** | **5,767 (52%)** | **2,196 (68%)** | **0.16** | **<0.01** |
| **Common-law marriage** | **542 (4.9%)** | **128 (4.0%)** | **<0.01** | **<0.01** |
| **Others** | **329 (2.9%)** | **78 (2.4%)** | **<0.01** | **<0.01** |
| **Race** |  |  |  |  |
| **White** | **4,141 (37%)** | **1,117 (35%)** | **0.02** | **0.03** |
| **Mixed race** | **5,071 (45%)** | **1,503 (46%)** | **0.01** | **0.02** |
| **Black** | **1,673 (15%)** | **543 (17%)** | **0.02** | **<0.01** |
| **Others** | **285 (2.6%)** |  | **<0.01** | **<0.01** |
| **Vaccination Status at delivery** |  |  |  |  |
| ***One dose Previous Pregnancy + One dose during pregnancy*** | | |  |  |
| ***BNT162b2-BNT162b2*** |  | **1,169 (36%)** |  |  |
| **CoronaVac-CoronaVac** | **̶̶̶̶̶** | **154 (4.8%)** |  |  |
| ***One dose Previous Pregnancy + Two doses during pregnancy*** | | |  |  |
| ***BNT162b2-BNT162b2-BNT162b2*** |  | **897 (28%)** |  |  |
| **CoronaVac-CoronaVac-BNT162b2** |  | **316 (9.8%)** |  |  |
| ***Two doses Previous Pregnancy + One dose during pregnancy*** | | |  |  |
| ***BNT162b2-BNT162b2-BNT162b2*** | **̶̶̶̶̶** | **78 (2.4%)** |  |  |
| **CoronaVac-CoronaVac-BNT162b2** | **̶̶̶̶̶** | **623 (19%)** |  |  |
| **Unvaccinated** | **11,170 (100%)** | **̶̶̶̶̶** |  |  |
| ***Outcomes*** |  |  |  |  |
| **Small for gestational Age** | **725 (6.5%)** | **220 (6.8%)** |  |  |
| **Low weight (<2.5 kg)** | **1,019 (9.1%)** | **303 (9.4%)** |  |  |
| **Preterm birth (<37 weeks)** | **1,320 (12%)** | **394 (12%)** |  |  |
| **Apgar <7†** | **110 (1.0%)** | **32 (1.0%)** |  |  |
| **Neonatal Death (Up to 28 days)** | **76 (0.7%)** | **20 (0.6%)** |  |  |

**Table S5. Neonatal adverse outcomes hazard ratio in women vaccinated before pregnancy with any vaccine type and number of doses**

| **Outcome** | **HR (95% CI)** | **Schoenfield residuals (p-value)** |
| --- | --- | --- |
| Preterm | 1.04 (0.93 to 1.19) | 0.35 |
| SGA | 1.02 (0.87 to 1.26) | 0.9 |
| LBW | 1.03 (0.89 to 1.22) | 0.58 |
| Low apgar (<7) | 1.11 (0.70 to 1.79) | 0.27 |
| Death | 1.02 (0.59 to 1.88) | 0.14 |
